# Testosterone treatment combined with exercise to improve muscle strength, physical function and quality of life in men affected by inclusion body myositis: A randomised, double-blind, placebo-controlled, crossover trial

**DOI:** 10.1371/journal.pone.0283394

**Published:** 2023-04-11

**Authors:** Sophia G. Connor, Timothy J. Fairchild, Yvonne C. Learmonth, Kelly Beer, Ian Cooper, Glenn Boardman, Shaun Y. M. Teo, Behnaz Shatahmasseb, Rui Zhang, Krystyne Hiscock, Jerome D. Coudert, Bu B. Yeap, Merrilee Needham

**Affiliations:** 1 Royal Perth Hospital, Perth, Western Australia, Australia; 2 Centre for Molecular Medicine & Innovative Therapeutics, Murdoch University, Murdoch, Western Australia, Australia; 3 Discipline of Exercise Science, Murdoch University, Murdoch, Western Australia, Australia; 4 Perron Institute of Neurological and Translational Sciences, Nedlands, Western Australia, Australia; 5 Research Development Unit, Fiona Stanley Hospital, Murdoch, Western Australia, Australia; 6 Department of Clinical Biochemistry, Pharmacology and Toxicology, PathWest Laboratory Medicine, QEII Medical Centre, Nedlands, WA, Australia; 7 Affinity Clinical Research, Nedlands, Western Australia, Australia; 8 Division of Medicine, The University of Notre Dame Australia, Fremantle, Western Australia, Australia; 9 Department of Endocrinology and Diabetes, Fiona Stanley Hospital, Murdoch, Western Australia, Australia; 10 Medical School, University of Western Australia, Crawley, Western Australia, Australia; 11 Department of Neurology, Fiona Stanley Hospital, Murdoch, Western Australia, Australia; IRCCS Medea: Istituto di Ricovero e Cura a Carattere Scientifico Eugenio Medea, ITALY

## Abstract

**Introduction:**

Inclusion body myositis (IBM) is the most commonly acquired skeletal muscle disease of older adults involving both autoimmune attack and muscle degeneration. As exercise training can improve outcomes in IBM, this study assessed whether a combination of testosterone supplementation and exercise training would improve muscle strength, physical function and quality of life in men affected by IBM, more than exercise alone.

**Methods:**

This pilot study was a single site randomised, double-blind, placebo-controlled, crossover study. Testosterone (exercise and testosterone cream) and placebo (exercise and placebo cream) were each delivered for 12 weeks, with a two-week wash-out between the two periods. The primary outcome measure was improvement in quadriceps isokinetic muscle strength. Secondary outcomes included assessment of isokinetic peak flexion force, walk capacity and patient reported outcomes, and other tests, comparing results between the placebo and testosterone arms. A 12-month Open Label Extension (OLE) was offered using the same outcome measures collected at 6 and 12-months.

**Results:**

14 men completed the trial. There were no significant improvements in quadriceps extension strength or lean body mass, nor any of the secondary outcomes. Improvement in the RAND Short Form 36 patient reported outcome questionnaire ‘emotional wellbeing’ sub-category was reported during the testosterone arm compared to the placebo arm (mean difference [95% CI]: 6.0 points, [95% CI 1.7,10.3]). The OLE demonstrated relative disease stability over the 12-month period but with a higher number of testosterone-related adverse events.

**Conclusions:**

Adding testosterone supplementation to exercise training did not significantly improve muscle strength or physical function over a 12-week intervention period, compared to exercise alone. However, the combination improved emotional well-being over this period, and relative stabilisation of disease was found during the 12-month OLE. A longer duration trial involving a larger group of participants is warranted.

## Introduction

Inclusion body myositis (IBM) is the most commonly acquired skeletal muscle disease associated with ageing, with men three times more likely than females to be diagnosed [1, 2]. The prevalence in Australia has been estimated to be between 14.9–50.5 per million population [3, 4]. The most severely affected muscle groups include the quadriceps, forearm finger flexors and swallowing muscles; and as the disease progresses, muscles are affected globally leading to progressive disability and dependency. Participants usually require the use of a walking aid by around 8 years after disease onset and a wheelchair after 10 years [1]. The aetiopathogenesis of disease is not fully understood, but the muscle pathology includes both muscle fibre degeneration (with protein accumulation including TDP-43, β-amyloid and p62), as well as autoimmunity directed towards muscle fibres (manifesting as T cell invasion of non-necrotic muscle fibres and major histocompatibility complex (MHC) up-regulation in muscle fibres). There are no specific disease-modifying treatments for IBM and it is poorly responsive to known immunosuppressive regimes [1].

Exercise has long been considered a vital component of the therapeutic approach in inflammatory myopathies, improving overall quality of life and assisting with maintenance and even improvement of strength and function [5, 6]. Some evidence indicates exercise may also have a direct immunomodulatory effect [7]. There have been multiple studies into the effect of aerobic and anaerobic exercise in IBM, usually physiotherapy-led or unsupervised home exercise programs, with most showing significant positive results, such as improved muscle strength [6, 8, 9].

Testosterone also improves muscle strength through increased erythropoiesis and protein synthesis leading to muscle hypertrophy [10]. A study in 1996 by Bhasin et al. demonstrated that the combination of testosterone supplementation and exercise training improved upper and lower limb skeletal muscle strength and performance to a greater extent than exercise alone in healthy young and middle-aged men [11]. A similar effect is seen in older men, with improvement in muscle performance seen even in the absence of significant increases in lean mass [12]. Testosterone combined with an exercise program (compared with testosterone or exercise alone) improved physical functioning and quality of life in elderly men with low testosterone concentrations, showing the potential benefits of combining these interventions [13]. However, it is not known whether similar beneficial effects would be seen in older men with muscle disease, particularly IBM. A previous study of oxandrolone, a 17α-alkylated androgen, versus placebo in 60 participants with IBM reported a borderline significant effect on whole- and upper body strength (p = 0.06 for whole body maximal voluntary isometric contraction testing (MVICT), p = 0006 for upper extremity MVICT, p < 0.001 for stair climbing) [14]. However this study was limited by differences in pre-intervention strength between those receiving treatment in comparison with those receiving placebo; and possible unintentional unblinding of treatment allocations. No previous studies have combined exercise training and testosterone supplementation utilising a crossover design to negate variability between individuals’ baseline strength. This is clinically relevant as an additive benefit would encourage a multimodal management strategy for men with IBM.

The purpose of this pilot study was to conduct a randomised double-blind, two-arm crossover trial to assess whether testosterone on a background of exercise training would further improve measures of muscle strength, physical function and quality of life in men affected by IBM over and above exercise alone. This study is the first to apply a combination of testosterone supplementation and exercise training in this disease. It was hypothesised that testosterone supplementation in male IBM patients undertaking prescribed exercise would improve muscle strength, physical function and quality of life compared to exercise alone.

## Participants and methods

### Study design

This trial was registered with the Australian and New Zealand Clinical Trials Registry (ACTRN12618000755235), and approved by the Human Research Ethics Committee at Murdoch University (approval number 2017/262). Data was collected at the Academic Medical Centre, Institute for Immunology and Infectious Diseases (IIID), Murdoch University.

**Fig 1:**
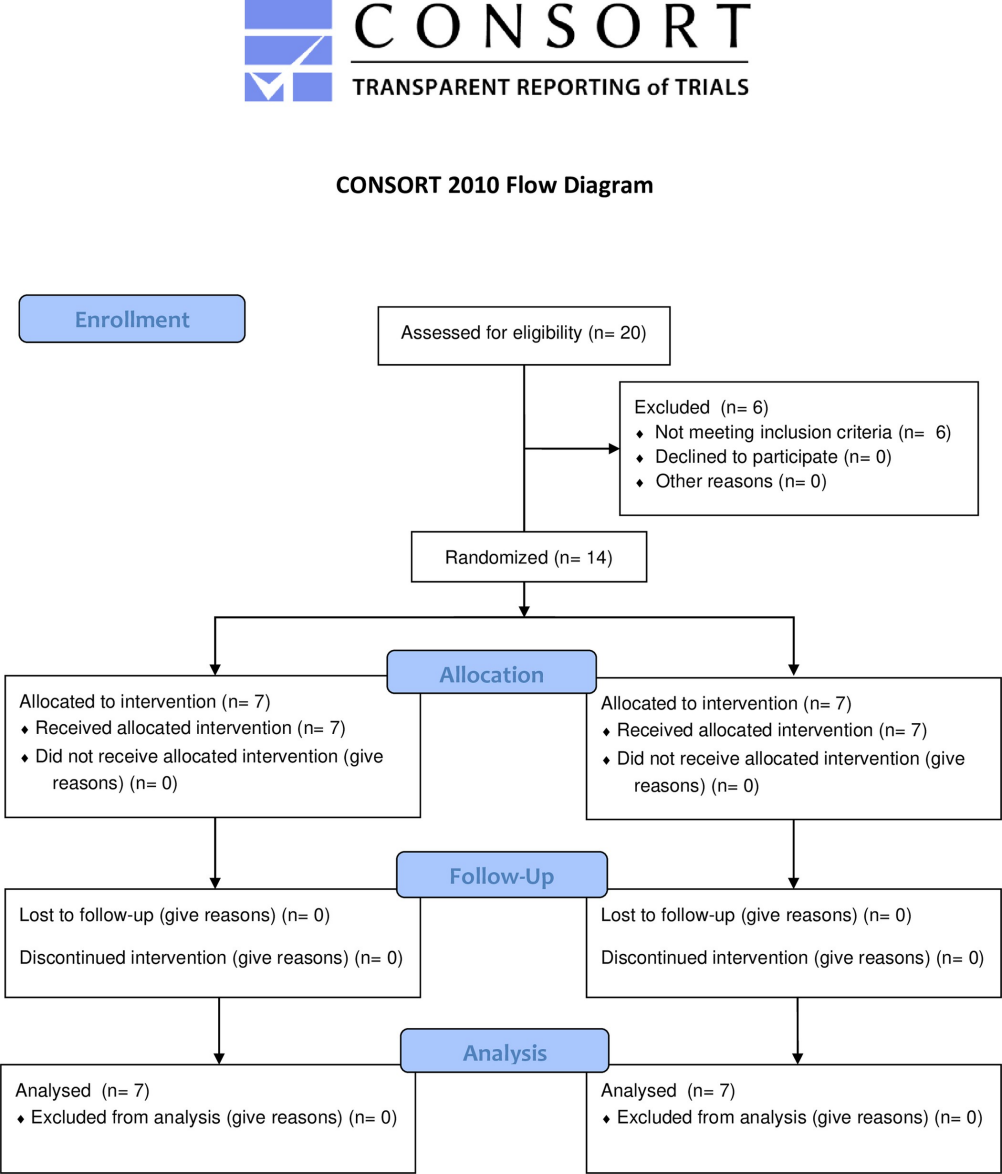
Trial design. All participants underwent randomisation and were allocated to either the placebo or testosterone arm.

The study design and methodology has been described within a previously published protocol [15]. The CONSORT checklist has been provided as (S1 Checklist). Informed written consent was obtained. In brief, a double-blinded, two-arm crossover randomised control trial was used to test whether testosterone added to exercise training would improve measures of muscle strength and physical function, as well as quality of life, in men affected by IBM. Within each of the two 12-week treatment arms, participants received either transdermal testosterone 100 mg (AndroForte5™ 50 mg/ml), applied to the torso every morning, or matching placebo. This was added to a prescribed, individualised exercise program spanning the entire study duration, with crossover to the alternate treatment (testosterone or placebo) after a two week ‘washout’ period (Weeks 12–14) (Fig 1). Transdermal testosterone at 100mg provides the standard therapeutic dosage for hypogonadal men [16] and has been shown in a previous randomised controlled trial to increase lean muscle mass in healthy middle to older aged men over a 12-week period [17].

Block randomisation was used to allocate treatment kits 1:1 to either testosterone or placebo at the central pharmacy. Randomised treatment kits were then allocated to participants in consecutive order at their Baseline visit (i.e. First participant enrolled received Treatment Kit no.1). Block size was determined by the central pharmacy. The random allocation sequence was generated by the Central Pharmacy (Lawley Pharmaceuticals). Participants were enrolled by the study PI and research nurses. Treatment kits (and therefore intervention) was assigned by allocation of the next available sequentially numbered Treatment Kit at site. Screening occurred between 10^th^ April 2018 and 16^th^ December 2018. The enrolment/randomisation date range was between the 19^th^ June 2018 and 18^th^ December 2018. Treatment allocations were not revealed until lock of the database for analysis.

The exercise training program was prescribed by an accredited exercise physiologist and accredited physiotherapist and comprised of light/moderate resistance training, balance training and moderate aerobic exercise component, as detailed in the protocol [15]. The exercise programs were tailored to. The individual’s functional and motor capabilities. Briefly, the training consisted of three resistance training (2 sets of 8 repetitions per exercise, 8–9 exercise per session; progressed to 3 sets of 12 repetitions by week 12) sessions per week, which was supplemented with additional aerobic exercise and balance/mobility program (e.g., walking, stationary cycling) for those able to perform these activities. The programs were standardised by prescribing a similar volume of work per muscle group between individuals, but individualized by modifying the intensity (5–7 on a modified 10-point rating of perceived exertion scale; associated descriptors: “last repetition is still easy”–“last repetition is more difficult than others”) or complexity of the movement (i.e., quadriceps contractions versus quadriceps extensions) and different progression trajectories. The duration of each exercise session was 30–60 minutes. Progression of exercises and adherence were monitored using training diaries and during phone calls throughout the intervention.

The pilot study was conducted as a double blind study. Participants, care providers and study team members were blind to intervention until unblinding of the allocations following database lock. An unblinded safety monitor provided review of safety bloods.

### Outcome measures

Outcome measurements were performed at the baseline, placebo (either week 12 or 26) and testosterone (either week 12 or 26) arms. The primary outcome measure was improvement in quadriceps isokinetic muscle strength assessed using a HUMACNORM isokinetic dynamometer (Stoughton, Massachusetts, USA) on the right leg at 30 and 60 degrees from full extension (0 degrees). Many studies have demonstrated inter-test reliability using the HUMACNORM isokinetic dynamometer [18–20]. Secondary outcomes included HUMACNORM isokinetic peak flexion force at 30 and 60 degrees, 30-second sit-to-stand test, timed up and go (TUG), walk capacity (2-minute walk test, 2MWT), 10-metre walk test (10MWT; 4 trials, two at self-selected pace and two at fast pace), hand-grip strength; and static and dynamic balance tasks (Berg Balance Scale, 4-stage balance test and Functional Reach). Further, during the 10MWT, participants walked across a 4.6m GAITRite (CIR systems Inc., New Jersey, USA) walkway [21]. The functional ambulation profile (FAP) score, velocity (cm/second), cadence (steps/minute), step length (cm), step time (seconds), base of support (cm) and double support were recorded and averaged. Dual Energy X-ray Absorptiometry (DXA) was utilised for assessment of lean body mass [22–26].

These secondary outcomes were used as they are commonly employed in the clinical and research setting. The Berg Balance Scale (BBS) is used to assess balance through a series of static and dynamic activities. The BBS has high inter-rater reliability (intraclass correlation coefficient = 0.99) and test-retest reliability (intraclass correlation coefficient = 0.98) in older populations [27, 28]. The TUG also is a timed-measure of dynamic balance with large normative ranges in elderly cohorts [29]. The inter-rater reliability (r = 0.99) of the TUG is high, and test-retest reliability (r = 0.56) is moderate in older populations [30]. The 30s sit-to stand test has a good test-retest reliability (0.84 to 0.92) [31]. The functional reach test (FRT) is a continuous measure designed to measure the limits of stability [32]. The FRT has been identified as a reliable, valid and easy to administer tool for limits of stability in people with Multiple Sclerosis [33]. The 2MWT has been shown to be a valid alternative to the 6MWT to describe walking capability among patients with neuromuscular disease during clinical trials [33]. The test-retest reliability has been previously characterised by the intraclass correlation coefficient and found to be .82 (95% CI, .76-.87) [24].

Patient reported outcomes regarding physical function and quality of life were assessed using the IBM-Functional Rating Scale (IBM-FRS) and the RAND Short Form 36 (RAND-SF-36) respectively [34–37]. Analysis of the RAND-SF-36 questionnaire was via the eight health domains defined within the survey, which include physical functioning, role limitations due to physical health, role limitations due to emotional problems, energy/fatigue, emotional well-being, social functioning, pain and general health. The IBM-FRS is a clinician-administered ten-item survey which pertains to the impact of IBM on daily function and consists of 10 questions, each of which can be scored 0–4. The 10-items utilise a four-point scale with anchors of “normal” (prior to onset of IBM) to a measure of dependency for the task. All 10 items are tallied to create a total score out of 40. Finally, participants were asked to record their daily study medication dosing, exercise adherence and any adverse events via a study diary; with adverse events being reported.

No changes were made to primary or secondary outcomes once the trial had commenced. The OLE study utilised the main study endpoints. Some timepoint deviations occurred during the OLE due to due to Coronavirus-related disruption.

### Data analysis

Data analysis was conducted in R statistical program using the package ggplot2 [38]. The independent variable was within subject. The dataset was tested formally for normality with the Shapiro test and visually using Q-Q normal plots. Data was analysed using paired t tests, or paired Mann-Whitney tests where normality assumption were in doubt. Outcome variables were adjusted for baseline variability through analysis of covariance (ANCOVA). A significant p value was considered as < 0.05. 95% confidence intervals were separately estimated by bootstrapping using 10000 repetitions in each run. A Benjamini and Hochberg p value adjustment was applied to all tests. Participants with missing data points were excluded from the models.

## Results

### Participant characteristics

In total, 20 males were assessed for eligibility for the study and six were excluded as they did not meet the selection criteria (Fig 1). Two participants had high PSA on screening, two had concomitant medications and two were already on testosterone supplementation. The remaining 14 participants were aged between 49 and 81 years with a mean age of 68 years (Table 1). Thirteen of the participants reported their ethnicity as Caucasian, with one participant reporting mixed Asian and Caucasian ethnicity. Of the 14 participants enrolled, eight individuals had been diagnosed within five years, and the remaining six diagnosed more than 5 years prior. Seven of the participants enrolled had clinicopathologically-defined IBM and three had clinically-defined IBM according to the ENMC Criteria [39]. The remaining four participants enrolled had clinically confirmed diagnoses of IBM on clinic-serological grounds with consistent clinical picture, blood test results and neurophysiology, but no biopsy available. There were no losses in the study after randomisation.

**Table 1:** Participant characteristics at baseline.

Participant Characteristics (N = 14)	
**Age**	68 ± 10.48 (range 48–81)
**Disease Duration > 5 years**	6 (46)
**Caucasian**	13 (93)
**Mixed Caucasian / Asian**	1 (7)
**Unable to ambulate on commencement of study**	4 (29)
**Unable to ambulate on completion of study**	6 (43)
**Baseline Weight (kg)**	86.66 ± 17.12
**Baseline Height (m)**	1.80 ± 0.06
**Baseline BMI (kg/m** ^**2**^ **)**	26.50 ± 4.14
**Baseline Testosterone (nM)**	11.28 ± 4.02

Mean ± standard deviation or frequency (%) provided when applicable.

All enrolled participants completed all study timepoints, however not all outcome measures could be performed due to disease severity, progression or adverse events (for example, falls with injury interfered with the 2MWT). On completion of the 26-week pilot study, a 12-month Open-Label Extension (OLE) was conducted at the request of 12 study participants, with reassessment at 6 and 12-month timepoints. Two participants did not enrol in the open-label study due to self-report of undesirable side effects relating to mood and/or libido observed whilst on testosterone.

### Primary outcome

Differences between the groups are presented as mean differences along with 95% confidence intervals. There were no treatment-associated differences in quadriceps extension at 30 degrees in Newton meters (Nm) (mean difference [95% CI]: 4.69 Nm [-5.68,11.46]) and 60 degrees (2.31 Nm [-4.38, 6.84]) during the testosterone period versus the placebo period (Table 2).

**Table 2:** Changes in outcome measures across placebo and testosterone arms and the open label extension arm.

Measure	Number of participants in each arm (n)	Baseline (95% CI)	Placebo (95% CI)	Testosterone (95% CI)	Difference Placebo / Testosterone (95% CI)	Difference Placebo / Testosterone (p value)	Difference End of Study and OLE (95% CI)	Difference End of Study and OLE (p value)
**DXA Scan LMM Total (kg)**	*n = 11 for placebo and testosterone*. *n = 7 for OLE*.	50.5 (46.28,54.36)	51.5 (47.33,55.72)	51.9 (47.39,56.09)	0.30 (-0.77,1.38)	0.614	1.76 (0.67,2.78)	0.012
**Peak Torque Extension 30**^**o**^ **(Nm)**	*n = 13 for placebo and testosterone*. *n = 4 for OLE*.	22.1 (8.09,34.91)	17.9 (4.93,29.33)	22.6 (0.92,38.92)	4.69 (-5.68,11.46)	0.672	-1 (-8,6.29)	0.595
**Peak Torque Extension 60**^**o**^ **(Nm)**	*n = 13 for placebo and testosterone*. *n = 4 for OLE*.	32.4 (10.59,51.00)	27.8 (7.85,44.46)	30.1 (4.69,49.54)	2.31 (-4.38,6.84)	0.905	0.375 (-3.75,3.25)	0.014
**Peak Torque Flexion 30**^**o**^ **(Nm)**	*n = 13 for placebo and testosterone*. *n = 4 for OLE*.	56.5 (36.67,71.58)	53.4 (38.55,65)	56.3 (39.31,69.46)	2.92 (-1.69,7.76)	0.292	-2.125 (-7.13,3.13)	0.76
**Peak Torque Flexion 60**^**o**^ **(Nm)**	*n = 13 for placebo and testosterone*. *n = 4 for OLE*.	38.5 (21.76,53.00)	33.5 (18.31,46.69)	36.3 (24.23,46.22)	2.77 (-3.23,9.31)	0.542	-0.5 (-8.25,10.38)	0.219
**Four Stage Balance Test (sec)**	*n = 11 for placebo and testosterone*. *n = 8 for OLE*.	28.8 (22.09,36.76)	31.1 (25.33,38.5)	32.5 (26.00,40.25)	1.46 (-3.50,6.38)	0.587	**12.36 (2.09,21.83)**	0.102
**Berg Balance**	*n = 13 for placebo and testosterone*. *n = 8 for OLE*	34.2 (24.23,44.38)	36.8 (27.38,47.15)	35 (25.00,45.62)	-1.77 (-5.23,2.23)	0.475	**11.84 (1.39,21.23)**	0.452
**Functional Reach (cm)**	*n = 13 for placebo and testosterone*. *n = 10 for OLE*.	24.3 (19.69,29.00)	24.4 (20.27,28.31)	25.8 (20.86,30.48)	1.4 (-1.58,4.40)	0.393	5.78 (-3.65,13.81)	0.246
**Grip Right (kg)**	*n = 13 for placebo and testosterone*. *n = 8 for OLE*.	13 (7.15,17.54)	13.7 (8.81,17.62)	14.5 (9.00,19.15)	0.808 (-1.50,2.65)	0.823	1.75 (-2.75,4.75)	0.416
**Grip Left (kg)**	*n = 13 for placebo and testosterone*. *n = 8 for OLE*.	11.8 (6.15,16.46)	10.9 (6.23,14.77)	9.46 (4.84,12.84)	-1.46 (-3.26,1.15)	0.382	-2.88 (-5.88,1.00)	0.937
**Timed Up and Go (sec)**	*n = 12 for placebo and testosterone*. *n = 7 for OLE*.	14.8 (4.82,22.52)	10.6 (5.80,15.18)	11.1 (6.03,15.87)	0.805 (-0.97,2.39)	0.395	-0.24 (-1.45,1.30)	0.5
**2 Minute Walk Test (m)**	*n = 12 for placebo and testosterone*. *n = 8 for OLE*.	109 (73.50,145.00)	110 (74.2,148.9)	105 (69.6,141.5)	-2.58 (-19.25,14.16)	0.776	-2.5 (-17.50,15.00)	0.606
**30s Sit To Stand (number performed)**	*n = 12 for placebo and testosterone*. *n = 7 for OLE*.	5.31 (2.76,7.69)	5.25 (2.41,7.91)	5.92 (2.70,9.00)	0.833 (-2.33,3.58)	0.778	**-4 (-7.86,-0.29)**	0.945

Results are presented as raw number, p value and 95% confidence intervals (lower, upper). Bolded results have significant 95% CI. Placebo = placebo phase, when the participants were provided with the placebo (either 0–12 weeks or 14–26 weeks depending on the participant). Testosterone = testosterone phase, when the participans were provided with testosterone (either 0–12 weeks or 14–26 weeks depending on the participant). OLE = open label extension. Difference Placebo / Testosterone refers to the difference between the placebo and testosterone phase of the trial. Difference end of study and OLE refers to the difference between the week 26 and the OLE 12 months result; and is presented in 95% CI and p values. Between the placebo and the testosterone phase was a wash out period. Nm = Newton meters, sec = seconds, kg = kilograms, cm = centimetre, m = meter. As not all participants were able to perform all outcome measures, n refers to the number of individuals analysed in each arm.

### Secondary outcomes

There were no treatment-associated differences in lean body mass assessed by DXA (mean difference [95% CI]: 0.30 kg [-0.77,1.38]). The remainder of the secondary outcomes showed no treatment-associated differences (Table 2). Changes in safety bloods can be seen in Table 3. There were no concerning changes to safety bloods across the baseline, placebo and testosterone arms of the study.

**Table 3:** Safety blood results across baseline, placebo, testosterone arms and the OLE.

	Haemoglobin (g/L)					Haematocrit					Creatinine Kinase (U/L)					Total Cholesterol (mmol/L)					Testosterone (nmol/L)					PSA (ng/mL)				
Participant ID	Baseline	Placebo	Testosterone	6 Months	12 Months	Baseline	Placebo	Testosterone	6 Months	12 Months	Baseline	Placebo	Testosterone	6 Months	12 Months	Baseline	Placebo	Testosterone	6 Months	12 Months	Baseline	Placebo	Testosterone	6 Months	12 Months	Baseline	Placebo	Testosterone	6 Months	12 Months
**1002**	160	146	141	NA	NA	0.47	0.43	0.43	NA	NA	322	195	185	NA	NA	4.3	3.4	3.7	NA	NA	11	8.4	11.5	NA	NA	1.6	NA	NA	NA	NA
**1003**	143	149	154	160	160	0.45	NA	0.47	0.5	0.51	307	302	321	163	232	4.2	4.2	3.6	4.3	3.9	12.2	12.1	21	20	18	1.2	1.13	1.2	1.4	1.6
**1004**	144	155	150	146	154	0.42	0.46	0.43	0.42	0.45	505	371	490	585	1370	6.2	7.2	6.9	7.9	5.7	13.5	20.3	18.4	28	17	0.84	0.93	0.96	1.2	1.3
**1005**	156	158	161	162	173	0.46	0.48	0.5	0.49	0.51	661	797	406	425	619	6.5	6.5	5.6	5.2	5.7	12.4	11.1	40.3	11	15	1.5	1.4	1.3	2.1	1.8
**1006**	135	129	140	140	135	0.37	0.37	0.39	0.39	0.39	94	167	198	173	113	5.2	4.9	4.9	4.9	5.1	9.2	13	18.7	13	12	1.4	1.3	1.4	1.7	2.3
**1007**	150	155	160	169	**181**	0.45	0.46	0.48	0.5	**0.54**	150	222	128	167	NA	3.8	4	4.2	4.3	4.6	16.7	22.4	26.2	31	15	2.4	1.9	2.6	**4.3**	3
**1008**	126	132	139	135	NA	0.39	0.41	0.43	0.43	NA	163	176	246	110	NA	4	4.1	4.2	5.1	NA	18.7	14.5	32.4	34	NA	<0.01	<0.01	<0.01	<0.01	NA
**1009**	149	156	155	158	**173**	0.45	0.47	0.46	0.48	**0.51**	179	252	346	321	384	4.8	5	4.8	4.6	5.5	3.9	4.3	14.5	10	11	0.82	0.69	0.68	0.8	1.1
**1010**	155	146	161	NA	NA	0.46	0.41	0.45	NA	NA	920	737	NA	NA	NA	5.1	4.6	4.7	NA	NA	7.4	6	8.7	NA	NA	0.55	0.49	0.43	NA	NA
**1011**	151	142	140	175	141	0.49	0.47	0.46	0.54	0.44	215	341	380	201	232	4.1	3.1	3.3	3.2	3.1	12.9	11.8	17.8	15	12	5.1	3.3	3.5	3.6	4.4
**1012**	141	145	137	147	NA	0.41	0.44	0.41	0.44	NA	617	390	346	310	NA	6.4	6.5	5.8	6.9	NA	7.9	6.3	15.2	7.6	NA	1.5	1.4	1.4	1.4	NA
**1014**	145	144	149	160	NA	0.44	0.43	0.45	0.48	NA	268	426	215	276	NA	6.8	6.2	5.8	5.2	NA	15	20.2	48.2	**58**	NA	2.1	2	1.5	**4.5**	NA
**1015**	163	153	157	166	170	0.5	0.47	0.49	0.51	0.51	295	312	415	574	385	6.4	4.7	4.1	4.4	3.7	9.5	20	14.6	16	17	2.7	2.8	1.9	3.4	2.4
**1016**	137	141	138	148	148	0.44	0.45	0.44	0.47	0.46	159	163	157	183	188	3.5	3.3	3.2	3	3.3	7.6	39.3	12.7	24	4.6	1.7	1.8	1.5	2.3	2.4

NA = not available and is also utilised when an individual withdrew from the trial. The highlighted numbers are listed as adverse events. The trial has been split into baseline, placebo and testosterone arms. Placebo = placebo phase, when the participants were provided with the placebo (either 0–12 weeks or 14–26 weeks depending on the participant). Testosterone = testosterone phase, when the participans were provided with testosterone (either 0–12 weeks or 14–26 weeks depending on the participant). The 6 months and 12 months columns refers to the OLE 6 month and 12 month time periods.

### Patient reported outcome questionnaires

**Fig 2:**
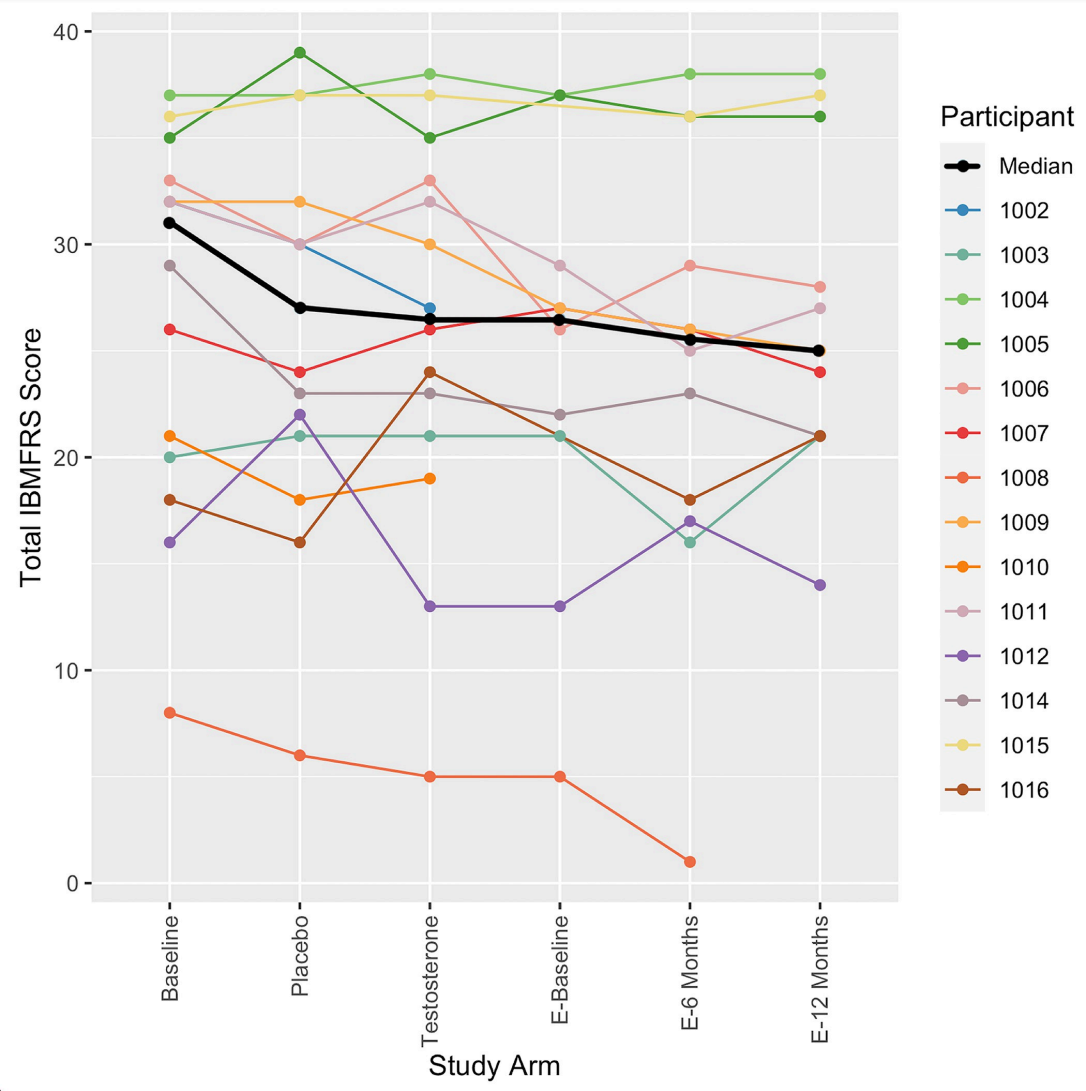
Change in IBM-FRS total score across the study period. The IBM-FRS consists of 10 questions, each of which can be scored 0–4. A score of 40 out of 40 indicates no impairment in function. This graph maps the change in total IBM-FRS score across the study arms. *E-baseline = OLE baseline result*, *E-6 months = OLE 6 months result*, *E-12 months = OLE 12 months result*.

Within the RAND-SF-36, there was a difference between testosterone and placebo groups in the domain of emotional wellbeing (mean difference [95% CI]: 6 [1.71:10.29]), with participants scoring higher in this domain (indicating better health status) during the testosterone arm compared to the placebo arm (Table 4). There were no differences in the other seven domains of the RAND-SF-36 questionnaire. The IBM-FRS questionnaire was analysed as total score out of 40, with a lower score indicating greater functional disability. Fig 2 shows the trend in IBM-FRS score over time for each individual participant. There were no significant differences observed between the testosterone and placebo arms (Table 4), but perhaps of interest, there was relative stability seen over the 12 month OLE, in contrast to the average 3-point decline expected in the natural history of IBM [34, 40, 41].

**Table 4:** Comparison of the RAND-SF-36 and IBM-FRS testosterone and placebo results.

Measure	Baseline (95% CI)	Placebo (95% CI)	Testosterone (95% CI)	Difference Placebo / Testosterone (95% CI)	Difference Placebo / Testosterone (p value)
**Physical Functioning**	32.1 (15.7,47.14)	36.8 (18.21,53.93)	33.6 (16.08,49.64)	-3.21 (-15.35,14.63)	0.754
**Role limitation due to physical health**	21.4 (7.14,33.93)	41.1 (19.64,60.71)	32.1 (12.50,51.79)	-8.93 (-32.14,17.86)	0.618
**Role limitation due to emotional problems**	57.1 (35.57,80.86)	78.6 (61.86,100.00)	76.1 (59.43,92.21)	-2.43 (-11.93,7.14)	0.571
**Energy/Fatigue**	45.4 (35.36,55.71)	43.2 (33.57,53.21)	50.7 (38.21,63.57)	7.5 (-1.07,15.71)	0.122
**Emotional Well-being**	20 (11.14,27.43)	17.7 (9.43,24.29)	23.7 (13.71,32.00)	**6 (1.71,10.29)**	**0.034**
**Social Functioning**	31.4 (13.50,46.64)	21.6 (10.93,32.36)	23.4 (9.00,35.93)	1.71 (-13.43,13.29)	1.000
**Pain**	38.5 (21.00,55.50)	29.1 (15.00,41.71)	31.1 (15.29,45.29)	1.93 (-7.36,10.93)	0.928
**General Health Perceptions**	62.1 (53.57,71.07)	58.6 (48.21,70.36)	59.3 (48.93,70.00)	0.714 (-8.21,9.29)	0.888

RAND-SF-36 *scores range from 0–100 where scores below 50 indicate worse quality of life than the population normative score and every 10 points indicates 1 standard deviation*. *Results are presented as raw numbers*, *p values*, *and 95% confidence intervals (lower*, *upper)*. *Bolded results have significant 95% CI or p values <0*.*05*. *Placebo = placebo phase*, *when the participants were provided with the placebo (either 0–12 weeks or 14–26 weeks depending on the participant)*. *Testosterone = testosterone phase*, *when the participans were provided with testosterone (either 0–12 weeks or 14–26 weeks depending on the participant)*. *Difference Placebo / Testosterone refers to the difference between the placebo and testosterone phase of the trial*.

### Adverse events

A high rate of participant adherence to the study protocol was verified through participant-reported medication and exercise diaries, combined with drug reconciliation processes. The most common adverse events (AEs) during the crossover period were falls and calf swelling, pain or cramping (Table 5), which were not considered directly related to testosterone; although 76% of falls occurred during the testosterone arm (total = 22 falls). There were only four instances of lower limb symptoms, three of which occurred in the testosterone arm but not considered related to the study. A majority of the AEs occurred during the testosterone arm (testosterone vs placebo, 25 events versus 6 events). During the OLE, 2 participants were withdrawn from the study due to elevated PSA (n = 1) or participant request (n = 1), however raised haemoglobin was noted at 12-month OLE timepoint for 2 participants (Table 3). Additional AEs were marked as possibly being associated with the study drug–upper and lower limb parasthesias, occurring in the testosterone phase; and weight gain, occurring in the placebo phase. In the OLE, the elevated PSA and elevated haemoglobin were considered probably related as they are known side effects from testosterone therapy [42]. The remainder were considered either as unrelated or unlikely related given the individual circumstances of each event.

**Table 5:** Adverse events dependent on trial arm.

Adverse Event	Placebo	Testosterone	OLE
**Fall**	4	13	9
**Calf swelling, pain or cramping**	1	3	0
**Weight Gain**	0	1	1
**Epistaxis**	0	1	0
**Muscle or Bone Injury**	1	3	2
**Dizziness or Vertigo**	0	1	3
**Urinary Retention**	0	1	0
**Upper and Lower Limb Parasthesias**	0	1	0
**Bradycardia**	0	1	0
**Increased Haematocrit**	0	0	2
**Increased PSA**	0	0	2
**Episodes of Anxiety**	0	0	1
**Nocturnal Erections**	0	0	1
**Total**	**6**	**25**	**21**

The adverse events for this study are separated into placebo, testosterone and OLE arms. Placebo = placebo phase, when the participants were provided with the placebo (either 0–12 weeks or 14–26 weeks depending on the participant). Testosterone = testosterone phase, when the participans were provided with testosterone (either 0–12 weeks or 14–26 weeks depending on the participant). OLE = open lable extension.

### Open label extension study

**Fig 3:**
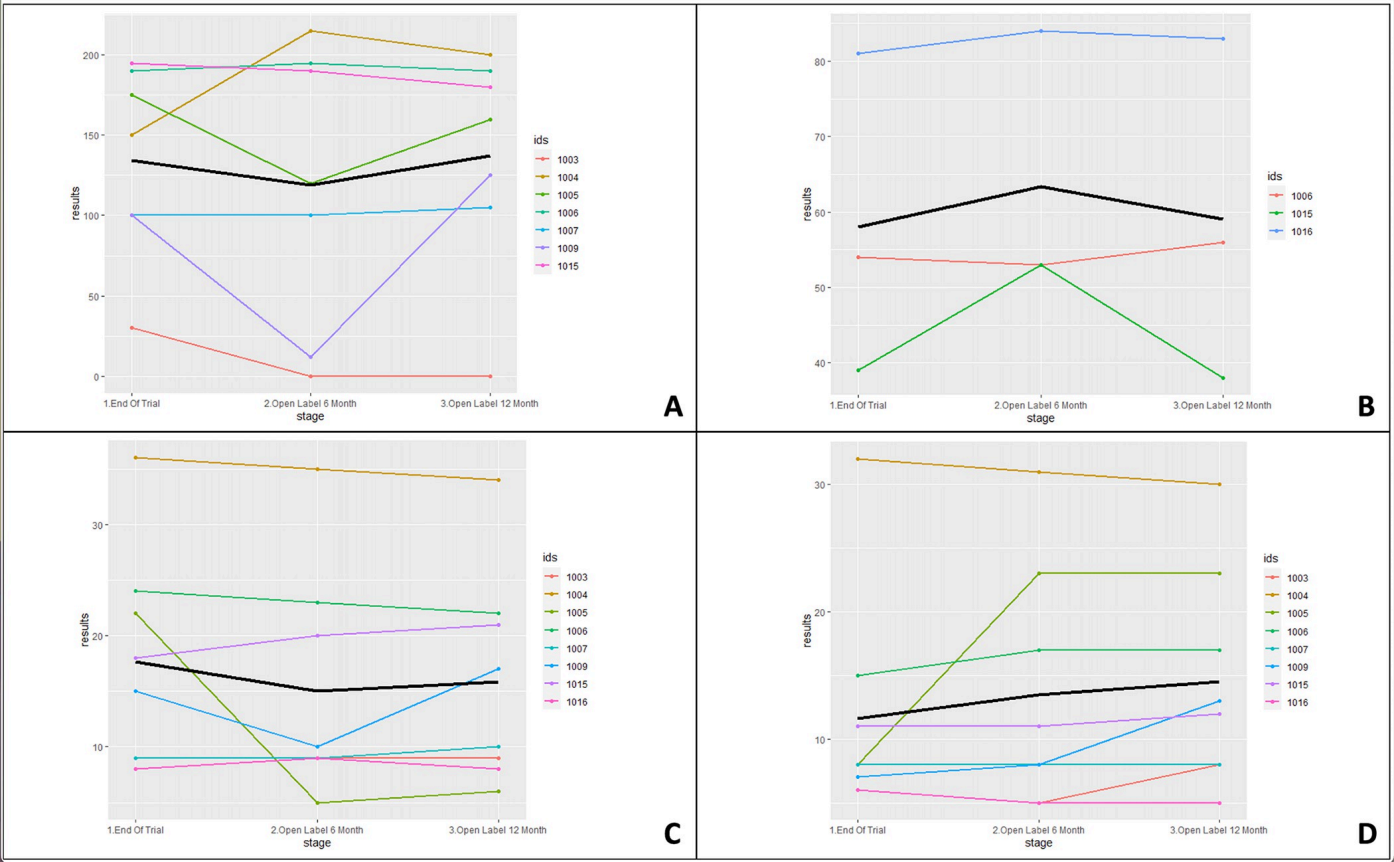
Results of the OLE study across 6 and 12 months. A summary of four of the secondary outcome measures separated into three timepoints—the end of the trial, 6 months and 12 months. The black line in the graphs refers to the average. Ids = participant IDs. **A**. Results of the 2MWT (m). **B**. Results of the peak torque extension at 60 degrees (Nm). **C**. Results of grip right strength (kg). **D**. Results of grip length strength (kg).

Two men declined to enter the OLE, leaving 12 participants. The results for the 2MWT, peak torque extension 60 degrees, right grip strength and left grip strength have been provided as graphs (Fig 3); and the OLE results are present in Table 2. The LMM (mean difference [95% CI]: 1.76 kg [95% CI 0.67:2.78]), four stage balance test (mean difference [95% CI]: 12.36s [2.09:21.83]) and Berg Balance test (mean difference [95% CI]: 11.84 [1.39:21.23]) improved across the OLE. The 30s sit-to-stand repetitions reduced across the OLE (mean difference [95% CI]: -4 repetitions [-7.86:-0.29]). IBMFRS and peak torque extension at 30 and 60 degrees were similar across the 12 month OLE, showing no significant decline (mean difference [95% CI]: -1 Nm [-8:6.29] and mean difference [95% CI]: 0.375 Nm [-3.75:3.25] respectively), although some participants were unable to perform this test due to the severity of quadriceps weakness. The safety blood results can be found in Table 3.

### Post- trial survey

This optional survey was answered by 71% of participants (n = 10) and their partners (n = 10). Of these, 70% of participants and 60% of their partners correctly determined when the participant was on the testosterone arm of the study. From the participant perspective, this was because of perceived changes in libido (n = 1), energy (n = 3) or both (n = 2), although one participant did not notice either of these. Partners also noted changes in libido (n = 1), energy (n = 1) or both (n = 1). The remainder did not report any changes (n = 3). Many of the participants (n = 6) and partners (n = 7) discussed these changes together which may have led to some unintentional unblinding.

## Discussion

In this 26-week pilot study comparing the combination of testosterone supplementation and exercise training versus exercise alone, we were unable to detect a significant additional effect of testosterone on muscle strength or physical function in men with IBM over the 12-week intervention period. However, individuals on testosterone had improved emotional wellbeing on the RAND-SF-36. Twelve men requested the OLE due to perceived positive effects of testosterone.

The emotional wellbeing aspect of the RAND-SF-36 refers to general mental health including questions related to psychological distress and well-being. In this study, there was an improvement in emotional wellbeing when on testosterone, which suggests it may have a positive influence on mood. Other aspects of the RAND-SF-36 scale did not change significantly between the two treatment periods, meaning that individuals did not feel that testosterone helped with physical functioning, social functioning, pain, energy or role limitations due to emotional / physical health.

The natural history of IBM is one of relentless decline with progressive loss of muscle mass, strength and worsening disability. Prior studies have shown an annual decline in the IBMFRS between 2.7–3.8 points per year [34, 40, 41], and a decline in quantitative muscle strength by 27.9% [40]. Therefore, the finding of relative stability of the IBMFRS and quantitative muscle testing of both grip strength and knee extension over the 12-month OLE is of interest. However, the higher rate of AEs across this period, particularly rises in PSA and haemoglobin, highlights the importance of close observation if this intervention is to be pursued.

The most commonly reported adverse events during the trial were falls. Unfortunately, due to the nature of IBM, falls can be a particularly common event independent of age and years since first symptoms [43]. None of these falls were identified as being due to testosterone, however there were a higher number of falls during the testosterone arm. This is unusual given that testosterone improved muscle mass in previous studies, albeit not in the IBM setting [11–13, 17]. Another study of older adults determined that testosterone concentrations are not associated with falls, therefore the falls that occurred are most likely to have been due to the underlying disease, and not related to testosterone [44]. It is also possible that testosterone supplementation increased levels of confidence, which may have led to increased activity and falls during this arm. This increase in confidence is possibly reflected in the significant emotional wellbeing result, but was not directly measured. It is recommended that further studies assessing the influence of testosterone in IBM also closely monitor falls, as well as confidence of individual participants, in an attempt to better quantify this relationship.

In the post study survey given to participants and their partners, 70% of participants and 60% of their partners correctly determined when the participant was on the testosterone arm due to perceived changes in libido and energy. This has the potential to unintentionally unblind the study and bias the results, but also highlights an important area for discussion with patients receiving testosterone therapy. Increases in libido and energy and mood changes are likely to impact patients’ relationships and this may in turn affect the acceptability of testosterone as a therapy. Future studies need to consider and potentially allow for the impact of testosterone on these domains within the study design.

Strengths of this study include the randomised study allocation and its double-blind crossover design. Many parameters were assessed to enable a more comprehensive analysis of the potential relationship between IBM and testosterone replacement therapy. Participant adherence was excellent throughout the crossover study with no participant drop out. Limitations include the small, heterogenous population of individuals with IBM. However this is a relatively large sample size for a single centre randomised trial given the prevalence of the disease within Australia and globally. Given that this is a relatively rare condition, individuals enrolled were at vastly different stages of disease progression. This posed a challenge in creation of homogenous exercise programmes and determining suitable outcome measures, as some individuals were wheelchair-bound or unable to participate in some physical assessments. The crossover trial design attempted to mitigate this issue, however a large multi-centre study would be better as it will allow for selection of a larger, more homogenous group of individuals with IBM with outcome measures better suited to their functional level. There is also the possibility that no significance was noted in this study because the 12-week duration of intervention was too short. Although 12 weeks should be sufficient in healthy populations to detect changes in the predetermined outcome measures adopted here, perhaps in an older population with muscle disease a longer duration study is required to be able to measure a significant difference. For trials in the future, it may be beneficial to perform a double blinded, randomised controlled trial over a longer duration (e.g. 6 to 12 months).

In conclusion, this pilot study did not suggest a significant additive effect of testosterone combined with exercise over 12 weeks to improve muscle strength or physical function in men with IBM. However, testosterone improved patient perceived emotional wellbeing; and may confer a stabilisation effect on specific measures muscle strength and physical function. The complete retention rate of the main study and the willingness of most participating men to enter an OLE suggests that larger and longer duration studies using testosterone are warranted and feasible in this population.

## Supporting information

S1 Checklist CONSORT 2010 checklist: Testosterone treatment combined with exercise to improve muscle strength, physical function and quality of life in men affected by inclusion body myositis: A randomised, double-blind, placebo-controlled, crossover trial. (PDF)
