# Correction: Testosterone treatment combined with exercise to improve muscle strength, physical function and quality of life in men affected by inclusion body myositis: A randomised, double-blind, placebo-controlled, crossover trial

**DOI:** 10.1371/journal.pone.0305919

**Published:** 2024-06-18

**Authors:** Sophia G. Connor, Timothy J. Fairchi, Yvonne C. Learmonth, Kelly Beer, Ian Cooper, Glenn Boardman, Shaun Y. M. Teo, Behnaz Shahathamassebi, Rui Zhang, Krystyne Hiscock, Jerome D. Coudert, Bu B. Yeap, Merrilee Needham

The eighth author’s name is spelled incorrectly. The correct name is: Behnaz Shahathamassebi.

An additional affiliation is missing for the eighth author. Behnaz Shahathamassebi is also affiliated with Department of Exercise Physiology and Sport Injuries and Corrective Movements, Faculty of Sport Sciences, Ferdowsi University of Mashhad, Mashhad, Iran.
